# Combination of Trace Metal to Improve Solventogenesis of *Clostridium carboxidivorans* P7 in Syngas Fermentation

**DOI:** 10.3389/fmicb.2020.577266

**Published:** 2020-09-25

**Authors:** Yi-Fan Han, Bin-Tao Xie, Guang-xun Wu, Ya-Qiong Guo, De-Mao Li, Zhi-Yong Huang

**Affiliations:** ^1^Tianjin Key Laboratory for Industrial Biological Systems and Bioprocessing Engineering, Tianjin Institute of Industrial Biotechnology, Chinese Academy of Sciences, Tianjin, China; ^2^National Technology Innovation Center of Synthetic Biology, Tianjin, China

**Keywords:** *Clostridium carboxidivorans* P7, syngas fermentation, higher alcohol, molybdate, solventogenesis regulation

## Abstract

Higher alcohols such as butanol (C4 alcohol) and hexanol (C6 alcohol) are superior biofuels compared to ethanol. *Clostridium carboxidivorans* P7 is a typical acetogen capable of producing C4 and C6 alcohols natively. In this study, the composition of trace metals in culture medium was adjusted, and the effects of these adjustments on artificial syngas fermentation by *C. carboxidivorans* P7 were investigated. Nickel and ferrous ions were essential for growth and metabolite synthesis during syngas fermentation by P7. However, a decreased dose of molybdate improved alcohol fermentation performance by stimulating carbon fixation and solventogenesis. In response to the modified trace metal composition, cells grew to a maximum OD_600__nm_ of 1.6 and accumulated ethanol and butanol to maximum concentrations of 2.0 and 1.0 g/L, respectively, in serum bottles. These yields were ten-fold higher than the yields generated using the original composition of trace metals. Furthermore, 0.5 g/L of hexanol was detected at the end of fermentation. The results from gene expression experiments examining genes related to carbon fixation and organic acid and solvent synthesis pathways revealed a dramatic up-regulation of the Wood–Ljungdahl pathway (WLP) gene cluster, the *bcs* gene cluster, and a putative CoA transferase and butanol dehydrogenase, thereby indicating that both *de novo* synthesis and acid re-assimilation contributed to the significantly elevated accumulation of higher alcohols. The *bdh35* gene was speculated to be the key target for butanol synthesis during solventogenesis.

## Introduction

To address the increasing and urgent demand for sustainable and clean energy production, the biosynthesis of alcohols such as ethanol and *n*-butanol using renewable resources has been receiving increased worldwide attention (Babu et al., 2017; Ullah et al., 2018; Kamani et al., 2019). Although the US Department of Energy has proposed that ethanol can be used as a major component for achieving the goals of renewable fuels, higher alcohols such as butanol and hexanol are less hygroscopic, contain a higher volumetric energy density, and are less water soluble and less volatile, thereby making these alcohols much more promising for use as “drop-in” biofuels (Phillips et al., 2015). Additionally, higher alcohols exhibit much more flexibility compared to that exhibited by ethanol. For example, butanol is also an important precursor used in the paint, polymer, and plastic industries (Kushwaha et al., 2020). Owing to the decreasing price of petroleum, renewable bio-alcohol research has become increasingly focused on higher alcohols rather than on ethanol.

*Clostridium carboxidivorans* bacteria utilize CO, CO_2_, and H_2_ via WLP (Kim et al., 2020). Alcohols are the only major end-metabolites produced during the HBE process (Hexanol-Butanol-Ethanol process) in this species (Fernández-Naveira et al., 2017). The bioconversion of the gaseous substrates occurs in two stages that include the acidogenic phase and the solventogenic phase (Gottwald and Gottschalk, 1985; Huang et al., 1985; Duürre, 2005). During acidogenesis, acetate and butyrate are the primary metabolites, and there is a dramatic decrease in pH. To survive in such harsh environments, solventogenic clostridia initiate solventogenesis, during which the acids produced earlier are consumed and alcohols become the main metabolic products.

To maximize product acquisition, considerable relevant research, including global transcriptional, proteomic, and metabolomics research, has focused on HBE fermentation analyses. However, the mechanism underlying the metabolic shift from the acidogenic to the solventogenic phase remains unclear (Janssen et al., 2010; Jang et al., 2014; Cheng et al., 2019; Kim et al., 2020). Another vital problem for traditional ABE fermentation is that this technology uses high-starch feedstocks or molasses as substrates, and these are costly and exert a large impact on the food economy (Jiang et al., 2015; Rezania et al., 2020).

Inorganic nutrients are considered to be one of the four categories of molecules that are required for extant life. These nutrients primarily consist of molecules of only a few atoms such as H_2_, N_2_, CH_4_, CO, and CO_2_ that were released from volcanic exhalations in ancient times. Some transition metal ions such as cobalt, nickel, and iron possess centers that contain sulfido, carbonyl, and other ligands are catalytically active and promote the growth of organic superstructures through the process of carbon fixation. This is the theory that describes the chemoautotrophic origin of life (Wächtershäuser, 2006). Based on this theory, it has been speculated that the active-site structures of the enzymes involved in carbon fixation in *C*. *carboxidivorans* are often reminiscent of minerals. According to previous research, the active sites of bifunctional CO dehydrogenase (CODH)–acetyl–ScoA synthase (ACS), a key enzyme in the WLP of acetogens, contains nickel (Alfano and Cavazza, 2020).

Similar to its autotrophic relatives, the *C. carboxidivorans* strain P7 relies on the WLP to transform CO and CO_2_ into acetyl-CoA that can be further metabolized into organic acids and alcohols (Drake et al., 2008; Bengelsdorf et al., 2018). Recently, trace metals have been reported to exert a large impact on cell growth and metabolite synthesis in *C. carboxidivorans* strain P7. Li et al. (2018) investigated the effects of zinc on syngas fermentation by the *C. carboxidivorans* strain P7, and they found that increasing the zinc concentration upregulated the expression of *fdh*II and *bdh* and led to enhanced cell growth and ethanol and butanol production. Using a statistical method, Shen et al. (2017b) found that a combination of trace metals used with variable-temperature cultivation could enhance alcohol synthesis during CO-rich off-gas fermentation by *Clostridium carboxidivorans* P7. In ATCC medium 1754, the optimum concentration of the trace metals was found to be 5-fold Ni^2+^, Co^2+^, SeO_4_^2+^, and WO_4_^2+^, 3.48-fold Cu^2+^, 0.55-fold MoO_4_^2+^, 0.5-fold Zn^2+^ and (NH_4_)(_2_)SO_4_⋅FeSO_4_⋅6H(_2_)O, and 44.32 mu M FeCl_3_⋅6H(_2_)O. The production of alcohol and organic acid changed from 2.16 g/L and 2.37 g/L to 4.40 g/L and 0.50 g/L, respectively, and the alcohol-to-product ratio increased from 47.7% to 89.8%. Shen et al. (2017a) reported that when using an optimized combination of trace metals, the production of alcohol and organic acid changed from 2.16 g/L and 2.37 g/L to 4.40 g/L and 0.50 g/L, respectively, and this increased the alcohol-to-product ratio from 47.7% to 89.8%. The effects of tungsten and selenium on C1 gas bioconversion by an enriched anaerobic sludge and microbial community were also studied by Chakraborty et al. (2020). To date, a number of studies have been have been performed to examine the effects of trace metals on C1 gas fermentation by *Clostridium* spp. Trace metals not only serve as essential metal cofactors for metalloenzymes in the WLP and alcohol synthesis pathways (Chen, 1995; Korkhin et al., 1998; Bender et al., 2011), but they also regulate gene expression to reprogram the intracellular metabolic network in solvent-producing clostridia and acetogens (Matson et al., 2010; Wu et al., 2015). There have been few reports regarding the effects of combined trace metals on regulation of gene expression during C1 gas fermentation.

In this study, *C. carboxidivorans* P7 was fermented under simulated industrial converter gas conditions (50% CO/35% CO_2_/15% H_2_) in a modified medium. By varying the Ni, Fe, Co, Se, and Mo concentrations, it was revealed that Ni and Fe acted as two vital trace metals required for growth and metabolite synthesis. The concentration of molybdate was the key inhibitor of biomass accumulation and alcohol production. Based on kinetic analysis of product synthesis and CO utilization, we propose that molybdate-restricted CO consumption can hinder acid re-assimilation, ultimately leading to a failure in the transition from acidogenesis to solventogenesis. Furthermore, based on the newly published complete genome sequence of *C. carboxidivorans* P7, we constructed gene expression profiles of the WLP and the ethanol, butanol, acetate, and butyrate synthesis pathways under different fermentation conditions, and our results revealed the regulatory mechanism of molybdate on these pathways and identified a number of potential key genes that are involved in solventogenesis.

## Materials and Methods

### Strain, Medium, and Cultivation

*C. carboxidivorans* P7 was generously provided by the laboratory of Professor Yang Gu (Li et al., 2015) and was maintained at −80°C with 20% glycerol for long-term preservation. For cultivation, frozen cells were revived in modified Wilkins Chalgren Anaerobic Medium (ATCC medium no. 2713) for 36 h and then transferred to fresh medium until the OD_600__nm_ reached 1.0, which served as the seed for gas fermentation. We modified Wilkins Chalgren Anaerobic Medium by replacing 10 g/L of gelatin peptone with 10 g/L of fish peptone. The gas fermentation medium was formulated on the basis of modified standard acetogen medium (Li et al., 2015; Huang et al., 2016). The pH of the medium was adjusted to 6.0 using solid NaOH.

### Preparation of Trace Element Solutions

Trace element solutions were prepared by mixing the reagents together in ddH_2_O. The concentrations of the trace metals in the original recipe are presented in Table 1, and the concentrations are defined as 1x. Additionally, five different trace element solutions that lacked either **Ni**Cl_2_⋅6H_2_O, (NH_4_)_2_⋅**Fe**SO_4_⋅6H_2_O, **Co**Cl_2_⋅6H_2_O, Na_2_**Se**O_4_, or Na_2_**Mo**O_4_⋅2H_2_O were prepared and have been defined as Ni (0x), Fe (0x), Co (0x), Se (0x), and Mo (0x), respectively. To determine the concentration of molybdate in the medium, the prepared medium was transported to SGS for quantitative analysis of Mo.

**TABLE 1:** Concentration of metals in the original medium (1x).

Trace metal	(μM)
(NH_4_)_2_⋅**Fe**SO_4_⋅6H_2_O	20.40
**Co**Cl_2_⋅6H_2_O	8.41
**Zn**SO_4_⋅7H_2_O	6.96
**Cu**Cl_2_⋅2H_2_O	1.49
**Ni**Cl_2_⋅6H_2_O	0.84
Na_2_**Mo**O_4_⋅2H_2_O	0.83
Na_2_**Se**O_4_	1.06
Na_2_**W**O_4_⋅2H_2_O	0.61

### Syngas Fermentation in a Serum Bottle and a Bioreactor

A serum bottle (120 mL) containing 20 mL of modified standard acetogen medium was used for gas fermentation. The headspace of the bottle was purged using 99.995% nitrogen for 5 min and then sealed with a rubber stopper. All prepared serum bottles were sterilized at 121°C for 20 min. For gas fermentation, 1 mL of pre-cultured seed liquor was inoculated. The atmosphere within the headspace of the bottle was replaced with a simulated industrial converter gas (50% CO/35% CO_2_/15% H_2_) and pressurized to 0.2 MPa. Fermentation was performed at 37°C at an agitation speed of 200 rpm. Samples (1.5 mL) of the culture were acquired at 24-h intervals. The cell density, pH, metabolite concentration, and CO consumption were all analyzed. The gas atmosphere was replaced with fresh simulated industrial converter gas after each round of sampling to equalize the pressure. Gas fermentation experiments in serum bottles were performed in two batches. In each batch, three repeats were introduced. A 7-L bioreactor (New Brunswick, Eppendorf, United States) was used to investigate the effects of the novel trace metal composition under continuous gas-fed conditions. A 300-mL culture was inoculated in 3 L of fermentation medium, and fermentation was performed at 37°C with agitation at 200 rpm with a gas flow rate of 400 mL/min. Gas fermentation in the bioreactor was performed in two batches.

### Analytical Procedures

The pH was measured using an FE-20 FiveEasy Plus laboratory pH meter (METTLER TOLEDO, United States). The cell density, as represented by the optical density, was monitored using a UV-1800 ultraviolet spectrophotometer (SHIMADZU, Japan) at an OD of 600 nm. The ORP was measured using an S220 SevenCompact^TM^ pH/Ion Meter (METTLER TOLEDO, Switzerland). The fermentation products were measured according to an internal standard method (Huang et al., 2016) using gas chromatography (FULI GC-9790, Wenling, China) equipped with a flame ionization detector and a capillary column (Zebron ZB-WaxPlus, Phenomenex, United States). All samples were prepared by centrifugation at 12,000 × *g* at 4°C for 5 min followed by filtration of the supernatant with 0.22-μm filters. The analysis was performed using the following program: oven temperature, 150°C; injector temperature, 180°C; and detector temperature, 180°C.

**FIGURE 1:**
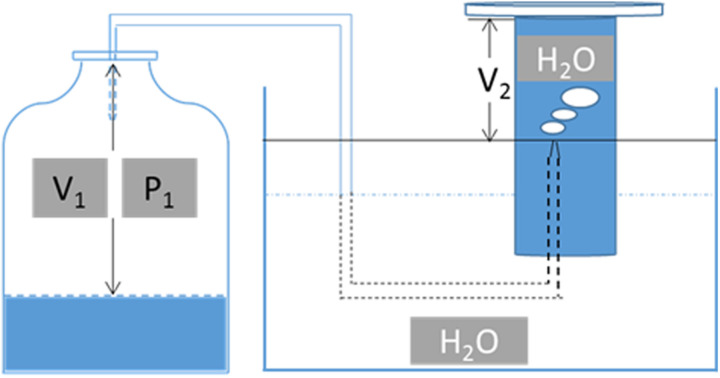
Schematic diagram for gas volume monitoring during fermentation. V1, the gas volume in the serum bottle, calculated by the total volume of the serum bottle minus the culture volume; P1, pressure in the serum bottle during gas fermentation; V2, the volume of H_2_O pushed out when transforming P1 into standard atmospheric pressure.

The CO concentration in the gas samples was analyzed using another gas chromatograph (Tianmei GC-7900, Shanghai, China) that was equipped with a TDX-01 80/100 mesh column (Techcomp, China) and a thermal conductivity detector. Helium was used as the carrier gas. The injector and detector temperatures were set to 80°C and 180°C, respectively, while the oven temperature was maintained at 150°C. The following equation was used to quantify CO based on the scheme presented in Figure 1:

$$n=\left(V1+V2\right)\times C_{0}$$

where *C*_0_ is the concentration of CO under standard atmospheric pressure as quantified by the standard curve method, *V*1 is the gas volume in the serum bottle as calculated by subtracting the culture volume from the total volume of serum bottle, *P*1 is the pressure in the serum bottle during gas fermentation, and *V*2 is the volume of H_2_O pushed out when transforming *P*1 into standard atmospheric pressure (Figure 1).

### Relative Gene Expression Profiles Detected by qPCR

Gene expression profiles were constructed for the Mo (0x) and Mo (1x) groups. Bacteria grown to mid-log phase (approximately 36 h of fermentation) were harvested using a Sorvall ST 16R centrifuge at 6,000 × *g* at 4°C for 5 min (Thermo Scientific, United States). The cell pellets were immediately frozen in liquid nitrogen and stored at −80°C. Total RNA was extracted using the Bacteria Total RNA Extraction kit (TIANGEN, Beijing, China) according to the manufacturer’s instructions. The RNA concentration and quality were determined using a Biophotometer Plus (Eppendorf, Hamburg, Germany) combined with gel electrophoresis analysis. Primers specific for 16S rDNA were used to test for DNA contamination in the total RNA samples (Metcalf et al., 2010). An aliquot (0.5 μg) of RNA was used to generate cDNA using the RevertAid First Strand cDNA Synthesis Kit (Thermo Scientific, Lithuania, EU) using random primers at 42°C for 30 min. cDNA (3 μL) and 0.3 μL of 20 μM gene-specific primers (Supplementary Table S1) were then mixed with the SYBR^®^ Select Master Mix (Applied Biosystems, Austin, TX, United States) to a final volume of 20 μL. The details of the detected genes are listed in Table 2, and these details are based on the genome accession number NZ_CP011803.1 (Li et al., 2015) and genomic analysis reported by Bruant et al. (2010). A LightCycler 96 real-time PCR system (Roche Diagnostics, Mannheim, Germany) was used to amplify and quantify the PCR products. The reaction mixtures were incubated for 5 min at 95°C, and this was followed by 40 amplification cycles of 10 s at 95°C and 30 s at 60°C. All reactions were performed in 96-well reaction plates. The transcript abundance of these genes was normalized to the housekeeping gene *guk* (formyl-tetrahydrofolate synthetase) that is constitutively expressed under the tested conditions (unpublished data). The relative transcript abundance levels of the studied genes were calculated using the 2^–△△CT^ method (Livak and Schmittgen, 2001). Fold changes of >2 or <0.5 were considered to be significant gene expression changes. Quantitative real-time PCR was performed in triplicate for each sample in every experiment, and the results were confirmed using biological duplicates.

**TABLE 2:** Targeted genes and relevant metabolic pathways.

Gene locus	Gene	Function	Pathway
Ccar_00570	*guk*	Guanylate kinase	Reference gene
Ccar_18835	*fhs*	Formate–tetrahydrofolate ligase	WLP gene cluster
Ccar_18790	*acsE*	CODH/ACS complex, methyltransferase subunit	
Ccar_18795	*acsC*	Corrinoid iron-sulfur protein large subunit	
Ccar_18800	*acsD*	Corrinoid iron-sulfur protein small subunit	
Ccar_18805	*CooCI*	Carbon monoxide dehydrogenase	
Ccar_18840	*CooCII*	Carbon monoxide dehydrogenase	
Ccar_18845	*acsA*	CODH/ACS complex, CODH subunit	
Ccar_18785	*acsB*	Acetyl-CoA decarbonylase/synthase complex subunit alpha/beta	
Ccar_18815	*metF*	Methylenetetrahydrofolate reductase	
Ccar_18825	*folD*	Methylene-tetrahydrofolate dehydrogenase/formyltetrahydrofolate cyclohydrolase	
Ccar_01740	*fdhI*	Formate dehydrogenase	CO_2_ reduction or formate oxidization
Ccar_16080	*fdhII*	Formate dehydrogenase	
Ccar_03945	*fdhIII*	Formate dehydrogenase	
Ccar_13505	*fdhIV*	Formate dehydrogenase	
Ccar_16050	*fdhV*	Formate dehydrogenase	
Ccar_22795	*bcd*	Butyryl-CoA dehydrogenase	Butyral-CoA synthesis from acetyl-CoA
Ccar_22780	*crt*	Crotonase	
Ccar_22785	*hbd*	3-Hydroxybutyryl-CoA dehydrogenase	
Ccar_22790	*thl*	Acetyl-CoA acetyltransferase	
Ccar_22800	*etfB*	Electron transfer flavoprotein	
Ccar_22805	*etfA*	Electron transfer flavoprotein subunit alpha	
Ccar_00690	*pta*	Phosphate acetyltransferase	Acetate synthesis from acetyl-CoA
Ccar_00695	*ack*	Acetate kinase	
Ccar_19520	*ptb*	Phosphate butyryltransferase	Butyrate synthesis from butyral-CoA
Ccar_19515	*buk*	Butyrate kinase	
Ccar_07995	*adh*	Acetaldehyde dehydrogenase	Alcohol synthesis from acetyl-CoA and butyral-CoA
Ccar_00050	*bdh50*	NAD(P)H-dependent butanol dehydrogenase	
Ccar_04610	*bdh10*	NADPH-dependent butanol dehydrogenase	
Ccar_24835	*bdh35*	NADPH-dependent butanol dehydrogenase	
Ccar_25840	*bdh40*	NADPH-dependent butanol dehydrogenase	
Ccar_01440	*CoAT*	CoA transferase	Acid re-assimilation

## Results

### Effect of the Absence of Nickel, Iron, Cobalt, Selenium, and Molybdenum on Cell Growth and Product Synthesis

**FIGURE 2:**
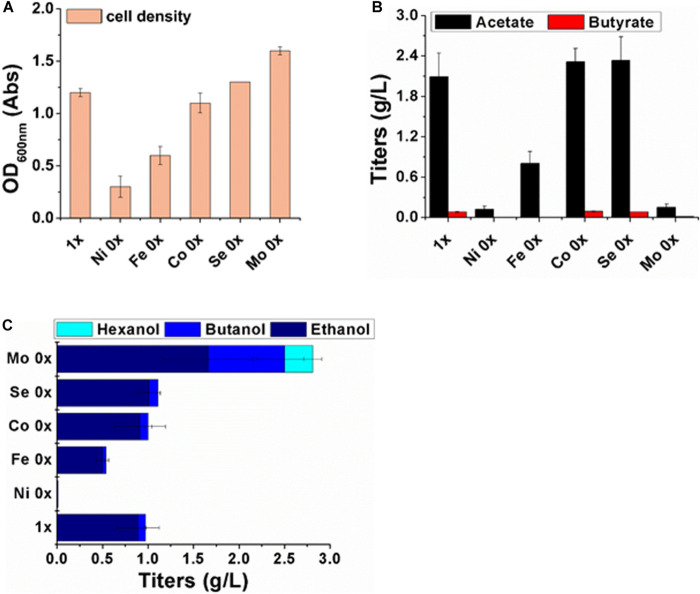
Cell growth and metabolites analysis after 3 days of fermentation with different metal compositions. **(A)** Cell density; **(B)** concentrations of acetate (filled black column) and butyrate (filled red column); **(C)** concentrations of ethanol (filled dark blue), butanol (filled blue) and hexanol (filled light blue).

The maximum OD_600__nm_ (1.2) of strain P7 was achieved in medium containing the full level of trace metals (Figure 2). The concentrations of the accumulated acetate and butyrate reached their maximum on day 3, and these concentrations were 2.09 g/L and 0.08 g/L, respectively. At the same time, the ethanol and butanol concentrations also reached their highest concentrations, and these were 0.89 g/L and 0.08 g/L, respectively. The hexanol titer was too low to be detected.

In the medium without Ni^2+^, evident deficiencies in both cell growth and metabolite synthesis were observed. The maximum acetate concentration was only 0.12 g/L, and no butyric acids and alcohols were detected. In the Fe (0x) medium, the values for the cell density and all of the product titers were reduced to approximately half of their values under the original medium conditions.

Without Co^2+^ and SeO_4_^2–^ supplementation in the medium, there were no significant effects on growth and product synthesis. In this study, molybdate was a key trigger for gas fermentation by strain P7. The actual concentration of Mo in the medium of Mo (0x) and Mo (1x) was analyzed by SGS test. There was 23ug/L of Mo in the Mo (0x) medium and 55 ug/L of Mo in the Mo (1x) medium (Supplementary Figures S1, S2). Of note, the cell density reached a maximum OD_600__nm_ of approximately 1.6 Abs in the Mo (0x) medium. Furthermore, the acetate and butyrate concentrations dramatically declined to 0.15 g/L and 0.05 g/L, respectively, while the ethanol and butanol yields were significantly increased to 1.66 g/L and 0.84 g/L, respectively. Additionally, 0.3 g/L of hexanol was produced on day 3, while the other treatments produced no detectable hexanol.

### Effect of Molybate on Cell Density, CO Utilization, and Metabolite Synthesis

Syngas fermentation was performed using three biological replicates under a 50% CO/35% CO_2_/15% H_2_ atmosphere. The response of *C. carboxidivorans* P7 toward molybdate was assessed by comparing cell growth, CO consumption, and metabolite synthesis in the Mo (0x) and Mo (1x) setups.

**FIGURE 3:**
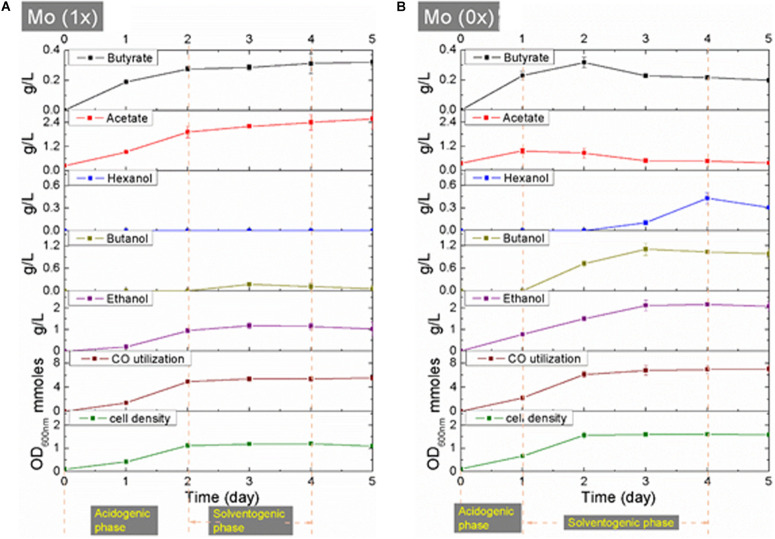
**(A,B)** CO utilization, cell growth and product synthesis during fermentation under Mo (0x) and Mo (1x).

Both cultures of *C. carboxidivorans* P7 in Mo (0x) and Mo (1x) media entered the stationary phase after two days of cultivation, and the maximum values of OD_600__nm_ were approximately 1.5 and 1, respectively (Figure 3). In the Mo (1x) group, acetate and butyric acid accumulated throughout the fermentation process and produced ethanol and butanol starting from days 2 and 3, respectively. The highest concentrations of acetate and butyric acid were 2.59 g/L and 0.32 g/L, respectively, at day 5. The accumulation of ethanol and butanol reached maximum levels of 1.19 g/L and 0.18 g/L, respectively, at day 3, and these levels then declined from day 4 to day 5 (Figure 3A). In the Mo (0x) group, the concentrations of acetic acid and butyric acid reached maximum levels (0.97 and 0.32 g/L, respectively) on day 1 and day 2, respectively, and these levels then began to decline. Ethanol was produced simultaneously with cell growth, while the synthesis of butanol and hexanol began on days 2 and 3, respectively. The peak concentration of ethanol was 2.17 g/L on day 4, while the maximum titer of butanol was 1.10 g/L on day 3. On day 4, the concentration of hexanol reached its maximum value of 0.43 g/L (Figure 3B).

To provide more insight into the differences between the two fermentation groups, the consumption of CO was investigated. In both groups, the utilization of CO was observed throughout the entire fermentation period, and the results are shown in Supplementary Table S2. The total consumption of CO was 29.06 mmol in the Mo (0x) group and 22.67 mmol in the Mo (1x) group (Supplementary Table S2), thereby indicating that more carbon was immobilized in the Mo (0x) group than that in the Mo (1x) group.

### Comparison of the Gene Expression Profiles of WLP During Syngas Fermentation Under Different Molybdenum Concentrations

**FIGURE 4:**
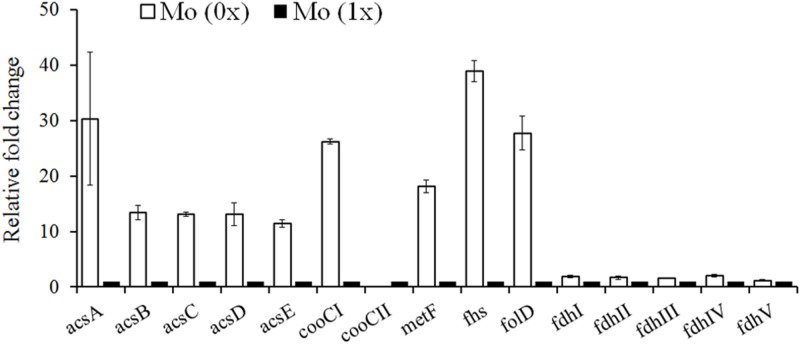
Gene expression profiles of the carbon fixation which was similar with Mo (1x) in this research. In our work, combination of trace metal could improve solvent generation only and shift of acidogenesis to solvent producing in strain p7 was observed. Ratio of solvent to acid was almost 20:1. Regulation of trace metal not only stimulated producing, but also was a trigger of acid reusing in strain p7. Lower concentration of acid in fermenting liquor could benefit downstream operation. acsA, CODH/ACS complex, CODH subunit; acsB, acetyl-CoA decarbonylase/synthase complex subunit alpha/beta; acsC, corrinoid iron-sulfur protein large subunit; acsD, corrinoid iron-sulfur protein small subunit; acsE, CODH/ACS complex, methyltransferase subunit; cooC, carbon monoxide dehydrogenase; fdh, formate dehydrogenase; fhs, formyl-tetrahydrofolate synthase; folD, bifunctional methylene-tetrahydrofolate dehydrogenase/formyl-tetrahydrofolate cyclohydrolase; metF, methylene-tetrahydrofolate reductase.

Based on the complete genome sequence information recently published by Li et al. (2015) and the genomic analysis performed by Bruant et al. (2010), the key enzymes responsible for acetyl-CoA synthesis from CO and CO_2_ were all recognized in the *C. carboxidivorans* P7 chromosome. We updated and remapped the WLP gene cluster (Table 2, and it ranged from Ccar_18775 to _18845 and contained 15 genes (Pierce et al., 2008; Bruant et al., 2010). Formate dehydrogenase (FDH), the enzyme responsible for CO_2_ reduction or formate oxidization, is not present in the WLP gene cluster. Five genes scattered throughout the genome were identified as FDH. These genes included Ccar_01740 (fdhI), Ccar_16080 (fdhII), Ccar_03945 (fdhIII), Ccar_13505 (fdhIV), and Ccar_16050 (fdhV), and they encode two selenocysteine-containing formate dehydrogenase H enzymes, a predicted formate dehydrogenase, a formate dehydrogenase accessory protein, and a predicted formate dehydrogenase D, respectively. We observed no significant difference in the expression levels of these five genes in cultures grown under Mo (0x) and Mo (1x) conditions (Figure 4). Additionally, we assessed the expression levels of 11 genes in the WLP gene cluster that were annotated and assumed to be functional enzymes in this pathway (Drake et al., 2008; Bruant et al., 2010). Among these genes, the transcripts of Ccar_18845 (cooCII) were nearly undetectable under both conditions. In the absence of Mo, the other 10 genes in the WLP were significantly upregulated compared to those in the presence of Mo (1x) (Figure 4).

### Comparison of the Gene Expression Profiles of the Metabolite Synthesis Pathways During Syngas Fermentation Under Different Molybdenum Concentrations

**FIGURE 5:**
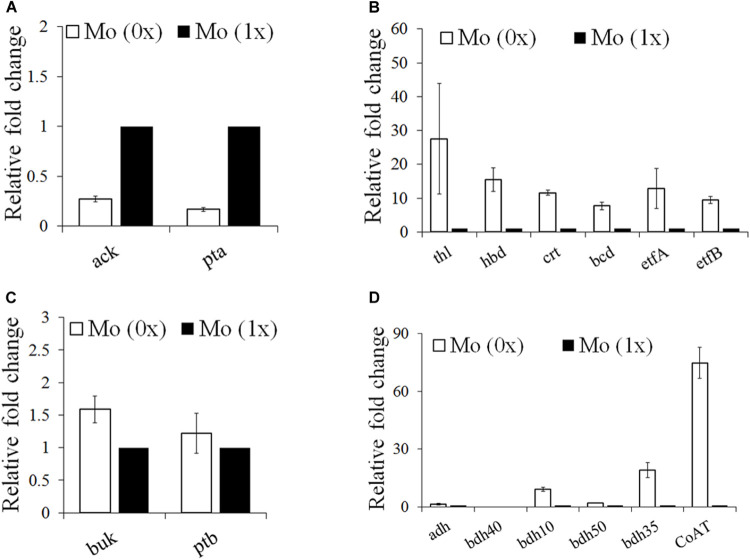
Gene expression profiles of the acids and butyryl-CoA and alcohol synthesis pathways under Mo (0x) and (1x). **(A)** Acetate synthesis from acetyl-CoA; **(B)** butyryl-CoA synthesis from acetyl-CoA; **(C)** butyrate synthesis from butyryl-CoA; **(D)** alcohol synthesis and acid re-assimilation related genes. ack, acetate kinase; adh, bifunctional acetaldehyde/alcohol dehydrogenase; bcd, butyryl-CoA dehydrogenase; bdh, butanol dehydrogenase; buk, butyrate kinase; pta, phosphate acetyltransferase; thl, acetyl-CoA acetyltransferase; hbd, 3-hydroxybutyryl-CoA dehydrogenase; crt, crotonase; etfA, electron transfer flavoprotein subunit alpha; etfB, electron transfer flavoprotein; ptb, phosphate butyryltransferase; CoAT, CoA transferase.

To elucidate the contrasting levels of gene expression and metabolite synthesis between the Mo (0x) and Mo (1x) conditions, we selected samples after 36 h of fermentation, as cultures at this point are at a stage of significantly different acid and alcohol syntheses. Based on information from a previous genome analysis (Li et al., 2015), we focused on genes involved in acetate (ack and pta), butyryl-CoA (crt, thl, and bcd), butyric acid (buk and ptb), ethanol (adh), and butanol (bdh) production. The expression levels of the acetate synthesis genes *ack* (Ccar_00695) and *pta* (Ccar_00690) were significantly downregulated in the Mo (0x) group (Figure 5A). Similar to the ABE fermentation performed by other Clostridia, *C. carboxidivorans* P7 converts acetyl-CoA into butyryl-CoA, which is the precursor for butyric acid and butanol synthesis, through *thl*, *hbd*, *crt*, *bcd*, *etfA*, and *etfB*. These six genes were clustered together on the chromosome from Ccar_22780 to Ccar_22805 and were all dramatically up-regulated under the Mo (0x) condition compared to levels observed in the Mo (1x) condition (Figure 5B). Two adjacent CDSs (Ccar_19515 and Ccar_19520) that encode *buk* and *ptb*, respectively, were involved in the conversion of butyryl-CoA to butyrate, and the expression levels of these genes exhibited no significant changes in response to alteration of the Mo concentration (Figure 5C). One CDS (Ccar_07995), present in the chromosome with two adjacent copies, was characterized as being involved in the ethanol and butanol production pathways. This gene encodes a bifunctional alcohol/acetaldehyde dehydrogenase that is involved in the conversion of acetyl-CoA to ethanol and butyryl-CoA to butanol. However, this *adh* gene exhibited no evident response to the change in Mo concentration (Figure 5D). In addition to *adh*, four *bdh* genes (*bdh50*, *bdh10*, *bdh40*, and *bdh35*) encoding a NAD(P)H-dependent butanol dehydrogenase and three different NADPH-dependent butanol dehydrogenases were also recognized on the P7 chromosome. Compared to levels in the Mo (0x) group, two of the NADPH-dependent butanol dehydrogenases (*bdh10* and *bdh35*) were significantly upregulated in Mo (1x). In particular, the expression level of *bdh35* exhibited a change of up to 20-fold, while the transcript of *bdh40* was too low to be detected (Figure 5D). In contrast, the expression level of the NAD(P)H-dependent butanol dehydrogenase *bdh50* did not change significantly under the Mo (0x) condition. Additionally, the predicted CoA transferase gene *CoAT* (Ccar_01440) that is speculated to be involved in the conversion of acetate and butyrate to acyl-CoA and butyryl-CoA, respectively, was dramatically up-regulated by 70-fold (Figure 5D).

### Syngas Fermentation in a 7-L Bioreactor With a Medium Containing Mo (0x)

**FIGURE 6:**
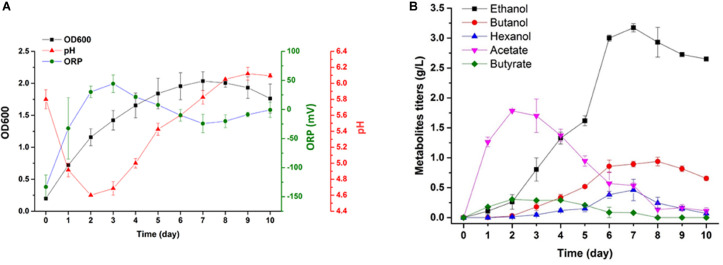
Cell growth, pH, ORP and product monitoring in a 7 L bioreactor under Mo (0x). **(A)** Cell density (filled black square), pH value (red triangle), and ORP value (green circle); **(B)** concentrations of acetate (inverted purple triangle), butyrate (green diamond), ethanol (black square), butanol (red circle) and hexanol (blue triangle).

Scaled-up syngas fermentation was performed using biological replicates (Figure 6). The highest cell density in the absence of Mo was 1.94. After 3 days of fermentation, bacteria entered the late log phase, and the acid concentration began to decrease until day 7. The pH values declined from day 0 to day 3. The maximum acetate and butyric acid concentrations (2 g/L and 0.3 g/L, respectively) were reached when the pH value was at the lowest point (∼4.6) (Figure 6). The alcohol yield increased significantly during the late log phase of growth, and the pH value also began to increase during this period. The highest pH value was 6.2. The highest ethanol, butanol, and hexanol yields were 3.2 g/L, 1 g/L, and 0.6 g/L, respectively (Figure 6B).

## Discussion

### Effects of Specific Trace Metals

In WLP, CODH, FDH, and H2ase that are involved in CO oxidation, CO_2_ reduction, and H_2_ oxidization, respectively, are all recognized as iron-sulfur proteins (Saxena and Tanner, 2011). Moreover, an iron-nickel-sulfur metal cluster was found to act as a critical component of the active sites of both CODH and ACS, which are responsible for acetyl-CoA synthesis. Oxidization of CO and H_2_ is the source of the reducing pool for acetogens under autotrophic conditions, and this process is not only tightly connected to ATP synthesis through the electron bifurcation mechanism or the cytochrome-based electron transport chain, but is also closely related to alcohol production via acetyl-CoA and butyryl-CoA reduction. Hence, the absence of nickel and iron should lead to a decrease in growth and metabolite synthesis, as reported in *C. ragsdalei* (Saxena and Tanner, 2011). However, the total cessation of cell growth under the Ni (0x) condition indicates that nickel is a key element for life in autotrophic solvent-producing clostridia.

Decreasing molybdenum concentrations dramatically promotes cell growth and alcohol synthesis, particularly for C4 and C6 alcohols. This negative regulation of alcohol synthesis by molybdenum is consistent with that mentioned in Shen’s report (Li et al., 2018). Moreover, molybdenum exerted no significant effect on cell growth or metabolite synthesis in *C. ragsdalei* (Saxena and Tanner, 2011), thereby indicating that the function of molybdenum was species-specific. In acetogens, Mo and/or tungsten are reported to serve as cofactors of FDH_H_, which is a key enzyme for catalyzing CO_2_ to formate conversion in WLP (Zhu and Tan, 2009). There are two types of FDH_H_ enzymes, including: molybdenum-containing formate dehydrogenase and tungsten-containing formate dehydrogenase. These two enzymes possess similar structures and functions. In strain P7, there might be a tungsten-containing formate dehydrogenase that lost its activity in Mo-supplemented medium (Fontecilla-Camps et al., 2009).

### Kinetic Analysis of Syngas Fermentation Under Different Molybdenum Concentrations

In solventogenic clostridia, C4 and C6 alcohols are generally synthesized through *de novo* synthesis and acid re-assimilation. *De novo* synthesis relies on the CoA-dependent Clostridium route (Zhang et al., 2012), while acid re-assimilation occurs via the function of carboxylic acid reductase or CoA transferase (Jones and Woods, 1986; Papoutsakis, 2008; Köpke et al., 2010). Time-course analyses of the acetate and butyrate concentrations revealed that removing molybdenum clearly reduced the acidogenic phase, activated acid re-assimilation, and prolonged the solventogenic phase. Moreover, cell growth and substrate utilization were also considerably increased. These results indicate that molybdenum may play a key role not only in affecting specific enzyme activity, but also in global metabolic networking in the gas fermentation process in *C. carboxidivorans* strain P7 (Alissandratos et al., 2013; Mintmier et al., 2020). In contrast, we expected that the external pH pressure and the concentration of undissolved organic acid would be two important factors for successful acid re-assimilation (Ramio-Pujol et al., 2015; Xie et al., 2015; Shen et al., 2017b). However, in this study, the external pH value was 5.1 when P7 shifted to acid-re-assimilation. This was much higher than the typical shifting point of ∼ 4.8, thereby indicating that the variation of trace metals was essential for metabolic shifting from acidogenesis to solventogenesis. Thus, it can be hypothesized that the intracellular metabolic model can be properly adapted to alcohol production by fine-tuning the trace metals within the medium of *C. carboxidivorans* P7.

In this study, the maximal productivity was 1.25g/L⋅d of ethanol, 0.47g/L⋅d of butanol, and 0.34g/L⋅d of hexanol in a 7-L CTRS bio-reactor. The total productivity of the solvent was 2.06g/L⋅d. According to previous research examining *C. carboxidivorans*, a maximal productivity of 2.5g/L⋅d of ethanol was achieved in a CTRS bio-reactor combined with monolithic packs for cell immobilization and 3.5g/L⋅d of ethanol in a CTRS bio-reactor coupled with a hollow fiber membrane. These accessories could immobilize cells and increase the mass transfer coefficient of gas to liquid. Although the productivity observed in these studies was higher than that reported by our study, 1.25g/L-1⋅d and 2.8g/L⋅d of acetate were also produced in their system (Shen et al., 2014a, b). The production of acid and solvent was simultaneous in their study, and this was similar to our observations using Mo (1x) in this study. In our work, a combination of trace metals could improve only solvent generation, and a shift from acidogenesis to solvent production in strain p7 was achieved. The solvent to acid ratio was nearly 20:1. Regulation of molybdenum concentration not only affected fermentation productivity, but also triggered acid reuse in strain p7. A lower concentration of acid in the fermenting liquor was found to benefit downstream operation.

### Effect of Different Molybdenum Concentrations on Gene Expression Profiles

**FIGURE 7:**
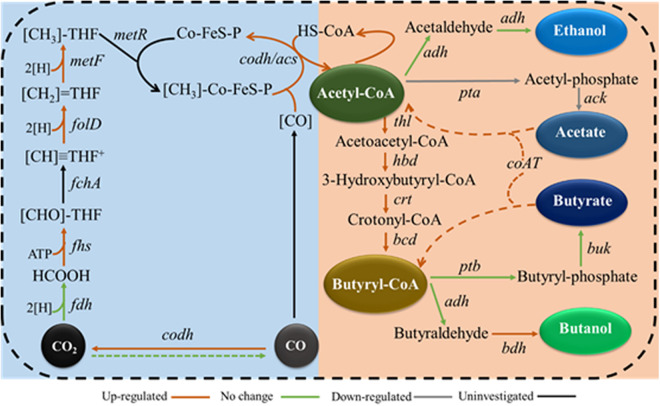
Hypothetical metabolic regulation model of molybdenum in *C. carboxidivorans* P7 during syngas fermentation. *codh*, carbon monoxide dehydrogenase; *fchA*, formimido-tetrahydrofolate cyclodeaminase; *metR*, methyltransferase; [H], from NADH or NADPH.

We expected molybdenum to act as a cofactor for FDH in a number of clostridia, and we also expected it to bind to tungsten active sites in enzymes. However, the regulatory role of FDH in the intracellular network in autotrophic solvent-producing clostridia remains unknown. Thus, the gene expression profiles of the WLP, acetate, butyrate, ethanol, and butanol synthesis pathways were examined to elucidate the regulatory role of molybdenum on syngas fermentation of *C. carboxidivorans* P7 and to identify potential key genes responsible for substrate utilization and solventogenesis. Compared to the Mo (1x) medium, the carbon fixation and butyryl-CoA synthesis pathways were significantly up-regulated when Mo was decreased in the Mo (0x) medium (Figure 6), and these findings are consistent with improved CO consumption and cell growth. The expression levels of the acetate synthesis genes *ack* (Ccar_00695) and *pta* (Ccar_00690) were found to be significantly downregulated in Mo (0x) medium, and this was consistent with the decrease in the acetate yield during 24 h of syngas fermentation. Among the three NADPH-dependent butanol dehydrogenases that were predicted to be highly related to solventogenesis, only *bdh10* and *bdh35* were significantly upregulated (especially *bdh35*), indicating that these two genes are potential key genes for higher alcohol production in *C. carboxidivorans* P7. Furthermore, expression of the predicted CoA transferase gene *CoAT* (Ccar_01440), that is thought to be involved in the transition from acetate and butyrate to acyl-CoA and butyryl-CoA, was dramatically up-regulated by 70-fold, thereby indicating an evident acid re-assimilation effect in low-concentration molybdenum medium. In summary, we hypothesize that a relatively low concentration of molybdenum leads to up-regulation of the WLP gene cluster, ultimately resulting in improved substrate utilization and acetyl-CoA accumulation. These factors, in combination with the down-regulation of the acetate and butyrate synthesis pathways and the up-regulation of solventogenesis-related genes, ultimately result in improved alcohol production (Figure 7).

### Elucidation of Carbon Destinations

Cell growth stagnated on day 4. The total amount of CO consumed on day 4 was re-calculated and found to be 22.04 mmol in the Mo (0x) group and 17.12 mmol in the Mo (1x) group. The total amount of carbon in the selected metabolites was 3.951 mmol in the Mo (0x) group and 3.02 mmol in the Mo (1x) group on day 4. The total amount of immobilized carbon was higher in the former; however, the proportion of carbon conversion from CO to metabolites was similar in the two groups, with 17.93% in the Mo (0x) group and 17.64% in the Mo (1x) group. Redundant fixed carbon flowed to form the cellular skeleton or to provide reducing power, and this was supported by data showing a higher cell density and titers of alcohol in the Mo (0x) group compared to the values in the Mo (1x) group. In regard to the converted carbon in the metabolites, approximately 87.12% was converted to produce alcohol in the Mo (0x) group, while only 37.36% was converted in the Mo (1x) group. A high ratio of alcohol in fermenter liquor could reduce the cost of the recovery process.

## Conclusion

In conclusion, Ni and Fe were found to be two vital metals for the growth of *C. carboxidivorans* P7 during syngas fermentation. Without the addition of these two metals, all product synthesis and biomass accumulation were significantly decreased. The dosage of molybdate was shown to be an important trigger for CO utilization and alcohol production, as this compound reduced the acidogenic phase, advanced the solventogenic phase, and dramatically increased alcohol production, particularly that of butanol and hexanol. Gene expression profiles of key metabolic pathways of *C. carboxidivorans* P7 under different syngas fermentation conditions were also determined in this study, and our results revealed that molybdate at 55 μg/L inhibited WLP and butanol synthesis pathways and the acid re-assimilation pathway. These results indicate that reducing this trace metal promotes alcohol production through both the *de novo* pathway and the acid re-assimilation mechanism.

## Data Availability Statement

The raw data supporting the conclusions of this article will be made available by the authors, without undue reservation.

## Author Contributions

Y-FH assisted in the experiment design, data analysis, and manuscript revising. B-TX designed and performed the experiments, analyzed the data, and drafted the manuscript, whose contributing was equally as first author. G-xW and Y-QG gave a hand in performing the experiments. Z-YH conceived the study and revised the manuscript. D-ML participated in the language editing. All authors read and approved the final manuscript.

## Conflict of Interest

The authors declare that the research was conducted in the absence of any commercial or financial relationships that could be construed as a potential conflict of interest.

## Abbreviations

Ni: nickel
Fe: iron
Mo: molybdenum
ORP: oxidation reduction potential
WLP: Wood–Ljungdahl pathway.

## References

[B1] Alfano M. Cavazza C. (2020). Structure, function, and biosynthesis of nickel-dependent enzymes. *Protein Sci.* 29 1071–1089. 10.1002/pro.3836 32022353 PMC7184782

[B2] Alissandratos A. Kim H. K. Matthews H. Hennessy J. E. Philbrook A. Easton C. J. (2013). *Clostridium carboxidivorans* strain P7T recombinant formate dehydrogenase catalyzes reduction of CO2 to formate. *Appl. Environ. Microbiol.* 79 741–744. 10.1128/Aem.02886-12 23144135 PMC3553769

[B3] Babu M. V. Murthy K. M. Rao G. A. P. (2017). Butanol and pentanol: The promising biofuels for CI engines A - review. *Renew. Sustain. Energy Rev.* 78 1068–1088. 10.1016/j.rser.2017.05.038

[B4] Bender G. Pierce E. Hill J. A. Darty J. E. Ragsdale S. W. (2011). Metal centers in the anaerobic microbial metabolism of CO and CO2. *Metallomics* 3 797–815. 10.1039/c1mt00042j 21647480 PMC3964926

[B5] Bengelsdorf F. R. Beck M. H. Erz C. Hoffmeister S. Karl M. M. Riegler P. (2018). Bacterial anaerobic synthesis gas (Syngas) and CO2+H2 fermentation. *Adv. Appl. Microbiol.* 103 143–221. 10.1016/bs.aambs.2018.01.002 29914657

[B6] Bruant G. Levesque M. J. Peter C. Guiot S. R. Masson L. (2010). Genomic analysis of carbon monoxide utilization and butanol production by *Clostridium carboxidivorans* strain P7(T). *PLoS One* 5:e13033. 10.1371/journal.pone.0013033 20885952 PMC2946384

[B7] Chakraborty S. Rene E. R. Lens P. N. L. Rintala J. Veiga M. C. Kennes C. (2020). Effect of tungsten and selenium on C-1 gas bioconversion by an enriched anaerobic sludge and microbial community analysis. *Chemosphere* 250:126105. 10.1016/j.chemosphere.2020.126105 32092562

[B8] Chen J. S. (1995). Alcohol dehydrogenase: multiplicity and relatedness in the solvent-producing clostridia. *FEMS Microbiol. Rev.* 17 263–273. 10.1111/j.1574-6976.1995.tb00210.x 7576768

[B9] Cheng C. Li W. Lin M. Yang S.-T. (2019). Metabolic engineering of *Clostridium carboxidivorans* for enhanced ethanol and butanol production from syngas and glucose. *Bioresour. Technol.* 284 415–423. 10.1016/j.biortech.2019.03.145 30965197

[B10] Drake H. L. Gossner A. S. Daniel S. L. (2008). Old acetogens, new light. *Ann. N. Y. Acad. Sci.* 1125 100–128. 10.1196/annals.1419.016 18378590

[B11] Duürre P. (2005). *Handbook on Clostridia.* Boca Raton, FL: Taylor & Francis.

[B12] Fernández-Naveira Á Veiga M. C. Kennes C. (2017). H-B-E (hexanol-butanol-ethanol) fermentation for the production of higher alcohols from syngas/waste gas. *J. Chem. Technol. Biotechnol.* 92 712–731. 10.1002/jctb.5194

[B13] Fontecilla-Camps J. C. Amara P. Cavazza C. Nicolet Y. Volbeda A. (2009). Structure-function relationships of anaerobic gas-processing metalloenzymes. *Nature* 460 814–822. 10.1038/nature08299 19675641

[B14] Gottwald M. Gottschalk G. (1985). The Internal-Ph of *Clostridium-acetobutylicum* and its effect on the shift from acid to solvent formation. *Arch. Microbiol.* 143 42–46. 10.1007/Bf00414766

[B15] Huang H. Chai C. Li N. Rowe P. Minton N. P. Yang S. (2016). CRISPR/Cas9-based efficient genome editing in *Clostridium ljungdahlii*, an autotrophic gas-fermenting bacterium. *ACS Synth. Biol.* 5 1355–1361. 10.1021/acssynbio.6b00044 27276212

[B16] Huang L. Gibbins L. N. Forsberg C. W. (1985). Transmembrane Ph gradient and membrane-potential in *Clostridium-acetobutylicum* during growth under acetogenic and solventogenic conditions. *Appl. Environ. Microbiol.* 50 1043–1047. 10.1128/aem.50.4.1043-1047.1985 4083872 PMC291790

[B17] Jang Y. S. Han M. J. Lee J. Im J. A. Lee Y. H. Papoutsakis E. T. (2014). Proteomic analyses of the phase transition from acidogenesis to solventogenesis using solventogenic and non-solventogenic *Clostridium acetobutylicum* strains. *Appl. Microbiol. Biotechnol.* 98 5105–5115. 10.1007/s00253-014-5738-z 24743985

[B18] Janssen H. Doring C. Ehrenreich A. Voigt B. Hecker M. Bahl H. (2010). A proteomic and transcriptional view of acidogenic and solventogenic steady-state cells of *Clostridium acetobutylicum* in a chemostat culture. *Appl. Microbiol. Biotechnol.* 87 2209–2226. 10.1007/s00253-010-2741-x 20617312 PMC3227527

[B19] Jiang Y. Liu J. Jiang W. Yang Y. Yang S. (2015). Current status and prospects of industrial bio-production of n-butanol in China. *Biotechnol. Adv.* 33 1493–1501. 10.1016/j.biotechadv.2014.10.007 25447782

[B20] Jones D. T. Woods D. R. (1986). Acetone-butanol fermentation revisited. *Microbiol. Rev.* 50 484–524. 10.1128/mmbr.50.4.484-524.1986 3540574 PMC373084

[B21] Kamani M. H. Es I. Lorenzo J. M. Remize F. Rosello-Soto E. Barba F. J. (2019). Advances in plant materials, food by-products, and algae conversion into biofuels: use of environmentally friendly technologies. *Green Chem.* 21 3213–3231. 10.1039/c8gc03860k

[B22] Kim J. Kim J. Um Y. Kim K. H. (2020). Intracellular metabolite profiling and the evaluation of metabolite extraction solvents for *Clostridium carboxidivorans* fermenting carbon monoxide. *Process Biochem.* 89 20–28. 10.1016/j.procbio.2019.10.012

[B23] Köpke M. Held C. Hujer S. Liesegang H. Wiezer A. Wollherr A. (2010). *Clostridium ljungdahlii* represents a microbial production platform based on syngas. *Proc. Natl. Acad. Sci. U.S.A.* 107 13087–13092. 10.1073/pnas.1004716107 20616070 PMC2919952

[B24] Korkhin Y. Kalb A. J. Peretz M. Bogin O. Burstein Y. Frolow F. (1998). NADP-dependent bacterial alcohol dehydrogenases: crystal structure, cofactor-binding and cofactor specificity of the ADHs of *Clostridium beijerinckii* and *Thermoanaerobacter brockii*. *J. Mol. Biol.* 278 967–981. 10.1006/jmbi.1998.1750 9836873

[B25] Kushwaha D. Srivastava N. Prasad D. Mishra P. K. Upadhyay S. N. (2020). Biobutanol production from hydrolysates of cyanobacteria *Lyngbya limnetica* and *Oscillatoria obscura*. *Fuel* 271:117583 10.1016/j.fuel.2020.117583

[B26] Li D. M. Meng C. X. Wu G. X. Xie B. T. Han Y. F. Guo Y. Q. (2018). Effects of zinc on the production of alcohol by *Clostridium carboxidivorans* P7 using model syngas. *J. Ind. Microbiol. Biotechnol.* 45 61–69. 10.1007/s10295-017-1992-2 29204741

[B27] Li N. Yang J. Chai C. Yang S. Jiang W. Gu Y. (2015). Complete genome sequence of *Clostridium carboxidivorans* P7(T), a syngas-fermenting bacterium capable of producing long-chain alcohols. *J. Biotechnol.* 211 44–45. 10.1016/j.jbiotec.2015.06.430 26193629

[B28] Livak K. J. Schmittgen T. D. (2001) Analysis of relative gene expression data using real-time quantitative PCR and the 2^−ΔΔ*C*_*T*_^ method. *Methods* 25, 402–408. 10.1006/meth.2001.1262 11846609

[B29] Matson E. G. Zhang X. N. Leadbetter J. R. (2010). Selenium controls transcription of paralogous formate dehydrogenase genes in the termite gut acetogen, *Treponema primitia*. *Environ. Microbiol.* 12 2245–2258. 10.1111/j.1462-2920.2010.02188.x 21966917

[B30] Metcalf D. Sharif S. Weese J. S. (2010). Evaluation of candidate reference genes in *Clostridium* difficile for gene expression normalization. *Anaerobe* 16 439–443. 10.1016/j.anaerobe.2010.06.007 20599622

[B31] Mintmier B. Nassif S. Stolz J. F. Basu P. (2020). Functional mononuclear molybdenum enzymes: challenges and triumphs in molecular cloning, expression, and isolation. *J. Biol. Inorgan. Chem.* 25 547–569. 10.1007/s00775-020-01787-y 32279136

[B32] Papoutsakis E. T. (2008). Engineering solventogenic clostridia. *Curr. Opin. Biotechnol.* 19 420–429. 10.1016/j.copbio.2008.08.003 18760360

[B33] Phillips J. R. Atiyeh H. K. Tanner R. S. Torres J. R. Saxena J. Wilkins M. R. (2015). Butanol and hexanol production in *Clostridium carboxidivorans* syngas fermentation: Medium development and culture techniques. *Bioresour. Technol.* 190 114–121. 10.1016/j.biortech.2015.04.043 25935391

[B34] Pierce E. Xie G. Barabote R. D. Saunders E. Han C. S. Detter J. C. (2008). The complete genome sequence of *Moorella thermoacetica* (f. *Clostridium thermoaceticum)*. *Environ. Microbiol.* 10 2550–2573. 10.1111/j.1462-2920.2008.01679.x 18631365 PMC2575129

[B35] Ramio-Pujol S. Ganigue R. Baneras L. Colprim J. (2015). Incubation at 25 degrees C prevents acid crash and enhances alcohol production in *Clostridium carboxidivorans* P7. *Bioresour. Technol.* 192 296–303. 10.1016/j.biortech.2015.05.077 26046429

[B36] Rezania S. Oryani B. Cho J. Talaiekhozani A. Sabbagh F. Hashemi B. (2020). Different pretreatment technologies of lignocellulosic biomass for bioethanol production: An overview. *Energy* 199:117457 10.1016/j.energy.2020.117457

[B37] Saxena J. Tanner R. S. (2011). Effect of trace metals on ethanol production from synthesis gas by the ethanologenic acetogen, *Clostridium ragsdalei*. *J. Ind. Microbiol. Biotechnol.* 38 513–521. 10.1007/s10295-010-0794-6 20694853

[B38] Shen S.H. Gu Y. Chai C. Jiang W. Zhuang Y. Wang Y. (2017a). Enhanced alcohol titre and ratio in carbon monoxide-rich off-gas fermentation of *Clostridium carboxidivorans* through combination of trace metals optimization with variable-temperature cultivation. *Bioresour. Technol.* 239:236. 10.1016/j.biortech.2017.04.099 28521234

[B39] Shen S. H. Gu Y. Chai C. S. Jihoang W. H. Zhuang Y. P. Wang Y. H. (2017b). Enhanced alcohol titre and ratio in carbon monoxide-rich off-gas fermentation of *Clostridium carboxidivorans* through combination of trace metals optimization with variable-temperature cultivation. *Bioresour. Technol.* 239 236–243. 10.1016/j.biortech.2017.04.099 28521234

[B40] Shen Y. Brown R. Wen Z. (2014a). Enhancing mass transfer and ethanol production in syngas fermentation of *Clostridium carboxidivorans* p7 through a monolithic biofilm reactor[J]. *Appl. Energy* 136 68–76. 10.1016/j.apenergy.2014.08.117

[B41] Shen Y. Brown R. Wen Z. (2014b). Syngas fermentation of *Clostridium carboxidivoran* P7 in a hollow fiber membrane biofilm reactor: Evaluating the mass transfer coefficient and ethanol production performance[J]. *Biochem. Eng. J.* 85 21–29. 10.1016/j.bej.2014.01.010

[B42] Ullah K. Sharma V. K. Ahmad M. Lv P. M. Krahl J. Wang Z. M. (2018). The insight views of advanced technologies and its application in bio-origin fuel synthesis from lignocellulose biomasses waste, a review. *Renew. Sustain. Energy Rev.* 82 3992–4008. 10.1016/j.rser.2017.10.074

[B43] Wächtershäuser G. (2006). From volcanic origins of chemoautotrophic life to bacteria, archaea and eukarya. *Philos. Trans. R. Soc. Lond. B Biol. Sci.* 361 1787–1808. 10.1098/rstb.2006.1904 17008219 PMC1664677

[B44] Wu Y. D. Xue C. Chen L. J. Wan H. H. Bai F. W. (2015). Transcriptional analysis of micronutrient zinc-associated response for enhanced carbohydrate utilization and earlier solventogenesis in *Clostridium acetobutylicum*. *Sci. Rep.* 5:16598. 10.1038/srep16598 26586044 PMC4653742

[B45] Xie B. T. Liu Z. Y. Tian L. Li F. L. Chen X. H. (2015). Physiological response of *Clostridium ljungdahlii* DSM 13528 of ethanol production under different fermentation conditions. *Bioresour. Technol.* 177 302–307. 10.1016/j.biortech.2014.11.101 25496952

[B46] Zhang J. Babtie A. Stephanopoulos G. (2012). Metabolic engineering: enabling technology of a bio-based economy. *Curr. Opin. Chem. Eng.* 1 355–362. 10.1016/j.coche.2012.09.003

[B47] Zhu X. F. Tan X. S. (2009). Metalloproteins/metalloenzymes for the synthesis of acetyl-CoA in the Wood-Ljungdahl pathway. *Sci. China Ser. B Chem.* 52 2071–2082. 10.1007/s11426-009-0082-3

