# Supplementary material for: Combination of Trace Metal to Improve Solventogenesis of Clostridium carboxidivorans P7 in Syngas Fermentation
Source: Front Microbiol. 2020 Sep 25;11:577266. doi: 10.3389/fmicb.2020.577266 (PMC7546793; doi:10.3389/fmicb.2020.577266)
Supplement: Supplementary file 1 [file Data_Sheet_1.docx]

**S 1. Primers for gene expression profiles analysis**

| **Gene locus** | **Primer** | **Sequence (5’- 3’)** | **Amplicon size (bp)** |
| --- | --- | --- | --- |
| Ccar_00570 | guk-RT-1 | AGGAGAAGTAGATGGCAC | 152 |
|  | guk-RT-2 | TGCCACTATCTACTGCTG |  |
| Ccar_18835 | fhs-RT-1 | TTACGCCAAGTTCCTTACAC | 193 |
|  | fhs-RT-2 | GGAGTTCCAAAGGCTGAT |  |
| Ccar_18790 | acsE-RT-1 | TTCTACCACCAGCATCAG | 197 |
|  | acsE-RT-2 | GGATTTCAACATAGGACCAG |  |
| Ccar_18795 | acsC-RT-1 | AGCAACACTTGCATCTTCAC | 151 |
|  | acsC-RT-2 | CTATGTAAGAATCGGCGAAG |  |
| Ccar_18800 | acsD-RT-1 | CCTAATGCTGCAAGTCTAAC | 200 |
|  | acsD-RT-2 | GCAGGTGCTATGGCTTAT |  |
| Ccar_18805 | CooCI-RT-1 | CTTCCTGCTCAAATGCTC | 119 |
|  | CooCI-RT-2 | GGCCTGGATGCTATTGT |  |
| Ccar_18840 | CooCII-RT-1 | GTATCAACTGTTCCCATCG | 199 |
|  | CooCII-RT-2 | CATTAGGGTTCCCACAAGA |  |
| Ccar_18845 | acsA-RT-1 | TCAGCATCTGCCATACAG | 154 |
|  | acsA-RT-2 | TCGGTAGACAAATGGGAG |  |
| Ccar_18785 | acsB-RT-1 | ACCTTATCCCAAATACCTGC | 179 |
|  | acsB-RT-2 | CTTTGTCAATCTTTCGCTCC |  |
| Ccar_18815 | metF-RT-1 | TGAACTCCGTCACAAAGA | 172 |
|  | metF-RT-2 | TAAAGTCAGCAGGTATGGC |  |
| Ccar_18825 | folD-RT-1 | AAGCAAGGTCACTTCCGT | 111 |
|  | folD-RT-2 | CAGATGCGATAAGTACGGT |  |
| Ccar_01740 | fdhI-RT-1 | AGGGAAGCAGTTAGGAATG | 175 |
|  | fdhI-RT-2 | CTATGGTATGGGAGTTTGTC |  |
| Ccar_16080 | fdhII-RT-1 | GTCTTTCGTAACTTGCTC | 139 |
|  | fdhII-RT-2 | GATGTAAAGCCTGATTGG |  |
| Ccar_03945 | fdhIII-RT-1 | AAGCAGTCGGCTGATGAT | 195 |
|  | fdhIII-RT-2 | TCCTGGTAAAGGCTCGTC |  |
| Ccar_13505 | fdhIV-RT-1 | TACTCTGCCGCTGAGTAT | 141 |
|  | fdhIV-RT-2 | GTTGCCCTTAGTGATACG |  |
| Ccar_16050 | fdhV-RT-1 | AGCGTCCTCGCATTGTCT | 184 |
|  | fdhV-RT-1 | TGTAGTAGCGGACCCAGT |  |
| Ccar_22795 | bcd-RT-1 | AGCAGCACAAGCATTAGGAC | 127 |
|  | bcd-RT-2 | CATTTCAGCTACCATCCAAG |  |
| Ccar_22780 | crt-RT-1 | AAACTCCAGAACAGGCAAGA | 123 |
|  | crt-RT-2 | ATTCACAACCGCCACCTA |  |
| Ccar_22785 | hbd-RT-1 | AGGTGCTAACCACCCAATA | 174 |
|  | hbd-RT-2 | CCTTTTCCAGTCTTTCTTCC |  |
| Ccar_22790 | thl-RT-1 | CATACTTTGCGGTTCAGG | 145 |
|  | thl-RT-2 | TCTTTGTCCCCATCTTCC |  |
| Ccar_22800 | etfB-RT-1 | TTCAGGTGCTAAGGTTACAG | 237 |
|  | etfB-RT-2 | TCTGGTCCTACTTGTGCTG |  |
| Ccar_22805 | etfA-RT-1 | ATGGCTGGGTAGACAAGG | 122 |
|  | etfA-RT-2 | GTCACTATCCTGCATTCC |  |
| Ccar_00690 | pta-RT-1 | GCGGTTAATCCATGTCCA | 128 |
|  | pta-RT-2 | GCACTTCCCATAGTTGAG |  |
| Ccar_00695 | ack-RT-1 | TCAGCAGTAGGTCACAGAGT | 174 |
|  | ack-RT-2 | CATTGGAGTGTTTGGCAT |  |
| Ccar_19520 | ptb-RT-1 | GCAGCAGCTTCTACAGAT | 126 |
|  | ptb-RT-2 | TCCTAGTATGCAGGCAAC |  |
| Ccar_19515 | buk-RT-1 | TCAACAACAACTGGGTCTAC | 116 |
|  | buk-RT-2 | GACCTAAAAGTTGGAGTGC |  |
| Ccar_07995 | adh-RT-1 | GAGTTCAACTAATCTTCCAGC | 112 |
|  | adh-RT-2 | GGAGCATTCAGAAGAGCC |  |
| Ccar_00050 | bdh50-RT-1 | TAACTTTAGCAGCAACGG | 101 |
|  | bdh50-RT-2 | AGGTGCCATATCAGGATG |  |
| Ccar_04610 | bdh10-RT-1 | GCAAGTGGATCAGAAAAGTG | 110 |
|  | bdh10-RT-2 | GCTCAAACAATGCCTCCTA |  |
| Ccar_24835 | bdh35-RT-1 | AAGCGGAACAGCTACAGA | 141 |
|  | bdh35-RT-2 | TTTGGTGGCATTGTTTGT |  |
| Ccar_25840 | bdh40-RT-1 | AATTGTTGTTGGAGGAGG | 130 |
|  | bdh40-RT-2 | AACGGTTTCAACTGATGG |  |
| Ccar_01440 | CoAT-RT-1 | CTGGAGGGATAGGTGGAGTA | 137 |
|  | CoAT-RT-2 | AGCCAAGCCTAAGAAAGC |  |

**S 2. CO quantification during fermentation under different concentration of MoO_4_^2-^**

| Time (day） | CO utilization (mmoles) | |
| --- | --- | --- |
|  | Mo (1x) | Mo (0x) |
| 0 | 0.00±0.00 | 0.00±0.00 |
| 1 | 1.42±0.20 | 2.21±0.19 |
| 2 | 4.93±0.28 | 6.10±0.49 |
| 3 | 5.39±0.35 | 6.79±0.77 |
| 4 | 5.38±0.35 | 6.94±0.68 |
| 5 | 5.55±0.36 | 7.02±0.74 |
| Total | 22.67 | 29.06 |
