# Supplementary material for: Combination of Trace Metal to Improve Solventogenesis of Clostridium carboxidivorans P7 in Syngas Fermentation
Source: Front Microbiol. 2020 Sep 25;11:577266. doi: 10.3389/fmicb.2020.577266 (PMC7546793; doi:10.3389/fmicb.2020.577266)
Supplement: Supplementary file 3 [file Image_2.pdf]

SGS

实验室编号:MNT1900783TJ
SGS报告编号:MCHTJ1900119-01
检测报告页码: 1 / 2

检 测 报 告

申请方名称: 中国科学院天津工业生物技术研究所
申请方地址: 天津空港经济区西七道32号
据称样品名称: M1
样品接收日期: 2019年1月25日
样品测试周期: 2019年1月25日 ~ 2019年1月30日
样品状态: 液体(189.4g)
样品编号: /

应申请方的申请，我实验室对申请方的样品依据相关标准或方法进行检测。具体检测结果见下页：

通标标准技术服务（天津）有限公司

杨宇红

授权签字人
2019年1月30日

敬告：此报告中涉及的样品由客户或按其指令执行的第三方所取得或提供。其结果仅严格与被检测样品相关，而不担保该样品对于任何货物具有代表性。我司不接受与样品的原产地或来源相关的任何责任。此检测报告仅用于客户科研、教学、内部质量控制、产品研发等目的，不具有社会证明作用，仅供内部参考。

Attention: To check the authenticity of testing /inspection report & certificate, please contact us at telephone: (86-755)83071443, or email: CN.Doccheck@sgs.com

通标标准技术服务（天津）有限公司 检验检测专用章 Inspection & Testing Services

该报告无授权签字人签名无效；未经我司书面批准，不得部分复制本报告。
此报告由我司依据其"服务通用条款"出具，请见网址 http://www.sgs.com/en/Terms-and-Conditions.aspx。请特别关注其中涉及责任限定，赔偿以及司法管辖的相关条款。
报告的持有方需知悉，此报告内容仅反映SGS在当时当地所得结论，且受限于客户指示。SGS仅对其客户负责，并且此报告不能免除交易各方根据交易文件所享有的权利和应履行的义务。对此报告内容及形式进行任何未经授权的修改，伪造或歪曲都是违法行为，违法者将会被追究法律责任。

Attention: To check the authenticity of testing /inspection report & certificate, please contact us at telephone: (86-755)83071443, or email: CN.Doccheck@sgs.com

检测项目	单位	检测结果	检测依据
		/	
Mo	µg/L	53	GB/T 30903-2014

备注: 1. 该样品非由本实验室采样，此报告不作为议付信用证使用。
2. 根据客户要求，该报告同时出具中英文报告，此份为其中文版本。仅中文版本具有法律效力。中英文版本如有歧异，请以中文版为准。

******结束******

该报告无授权签字人签名无效；未经我司书面批准，不得部分复制本报告。
此报告由我司依据其“服务通用条款”出具，请见网址 http://www.sgs.com/en/Terms-and-Conditions.aspx。请特别关注其中涉及责任限定，赔偿以及司法管辖的相关条款。
报告的持有方需知悉，此报告内容仅反映SGS在当时当地所得结论，且受限于客户指示。SGS仅对其客户负责，并且此报告不能免除交易各方根据交易文件所享有的权利和应履行的义务。对此报告内容及形式进行任何未经授权的修改，伪造或歪曲都是违法行为，违法者将会被追究法律责任。

SGS

Lab Reference No.:MNT1900783TJ
SGS Report No.:MCHTJ1900119-01
Testing Report Page: 1 / 2

TESTING REPORT

Declared Principal Name:	Tianjin Institute of Industrial Biotechnology, Chinese Academy of Science
Declared Principal Address:	Tianjin airport economic zone, west 7 Road, Tianjin, China
Declared Sample Name:	M1
Sample Received Date:	2019-01-25
Sample Testing Period:	2019-01-25 ~ 2019-01-30
Sample Condition on Receipt:	Liquid(189.4g)
Declared Sample Number:	/

In accordance with instructions received from applicant, we prepared and carried out required test on the sample. The analysis results reported as refer to the next page(s)

SGS-CSTC Standards Technical Services(TianJin) Co.,Ltd

Yuhong Yang

Authorised Signatory
2019-01-30

WARNING: The sample(s) to which the findings recorded herein (the "Findings") relate was(were) drawn and / or provided by the Client or by a third party acting at the Client's direction. The Findings constitute no warranty of the sample's representativeness of any goods and strictly relate to the sample(s). The Company accepts no liability with regard to the origin or source from which the sample(s) is/are said to be extracted.The test report shall only be used for clients' scientific research, teaching, internal quality control, product research and development, etc... and just for internal reference.

Attention: To check the authenticity of testing /inspection report & certificate, please contact us at telephone: (86-755)83071443, or email: CN.Doccheck@sgs.com

通标标准技术服务（天津）有限公司 检验检测专用章 Inspection & Testing Services

The report would be invalid without authorized signature. The report shall not be reproduced except in full, without written approval of the Company.
This document is issued by the Company under its General Conditions of Service accessible at http://www.sgs.com/en/Terms-and-Conditions.aspx. Attention is drawn to the limitation of liability, indemnification and jurisdiction issues defined therein.
Any holder of this document is advised that information contained hereon reflects the Company's findings at the time of its intervention only and within the limits of Client's instructions, if any. The Company's sole responsibility is to its Client and this document does not exonerate parties to a transaction from exercising all their rights and obligations under the transaction documents. Any unauthorized alteration, forgery or falsification of the content or appearance of this document is unlawful and offenders may be prosecuted to the fullest extent of the law.

Lab Reference No.:MNT1900783TJ

SGS Report No.:MCHTJ1900119-01

Test Items	Unit	Result	Standard No.
		/	
Mo	μg/L	53	GB/T 30903-2014

Remark: 1.The sample was not drawn by the laboratory and this report is not used for L/C negotiation.

2.Upon Client's request, this test report has been issued as the English version Only the Chinese version is a legally binding document and may be used for any legal purpose, In case of any discrepancy between Chinese version and English version, the Chinese version shall prevail.

******The end******

Attention: To check the authenticity of testing /inspection report & certificate, please contact us at telephone: (86-755)83071443, or email: CN.Doccheck@sgs.com

通标标准技术服务(天津)有限公司
检验检测专用章
Inspection & Testing Services

The report would be invalid without authorized signature. The report shall not be reproduced except in full, without written approval of the Company.
This document is issued by the Company under its General Conditions of Service accessible at http://www.sgs.com/en/Terms-and-Conditions.aspx. Attention is drawn to the limitation of liability, indemnification and jurisdiction issues defined therein.
Any holder of this document is advised that information contained hereon reflects the Company's findings at the time of its intervention only and within the limits of Client's instructions, if any. The Company's sole responsibility is to its Client and this document does not exonerate parties to a transaction from exercising all their rights and obligations under the transaction documents. Any unauthorized alteration, forgery or falsification of the content or appearance of this document is unlawful and offenders may be prosecuted to the fullest extent of the law.

SGS-CSTC Standards Technical Services (Tianjin) Co., Ltd.
Minerals service
No. 41, 5th Avenue, TEDA, Tianjin, China
Post Code:300457;Tel:(86 22)65288000;Fax:(86 22)25299577
www.sgsgroup.com.cn
e sgs.china@sgs.com

TJMIN 115148
